# Targeting Toll-like receptor 2 inhibits growth of head and neck squamous cell carcinoma

**DOI:** 10.18632/oncotarget.3393

**Published:** 2015-04-02

**Authors:** Lovisa Farnebo, Arash Shahangian, Yunqin Lee, June Ho Shin, Ferenc A. Scheeren, John B. Sunwoo

**Affiliations:** ^1^ Division of Head and Neck Surgery, Department of Otolaryngology, Stanford University School of Medicine, Stanford, CA, USA; ^2^ The Netherlands Cancer Institute, The Netherlands; ^3^ Stanford Cancer Institute and the Institute for Stem Cell Biology and Regenerative Medicine, Stanford University, Stanford, CA, USA

**Keywords:** head and neck cancer, inflammation, tumorigenesis

## Abstract

Infection-driven inflammation has been proposed to be involved in the tumorigenesis of head and neck squamous cell carcinoma (HNSCC). Oral HNSCC is often colonized with microbes such as gram-positive bacteria and yeast, where ligands derived from their wall components have been shown to specifically bind to Toll-like receptor 2 (TLR2). Although TLR2 has been described to be expressed in oral HNSCC, its function has not been well characterized. Here, we show the expression of TLR2 in both HNSCC cell lines and primary patient-derived HNSCC xenograft tumors. Activation of TLR2 with a yeast-derived ligand of TLR2, zymosan, promoted organoid formation in an *ex vivo* model of tumor growth, while blockade with anti-TLR2 antibodies inhibited organoid formation. Zymosan also induced phosphorylation of ERK and the p65 subunit of NF-κB, which was inhibited in the presence of anti-TLR2 antibodies, indicating that this receptor is functional in HNSCC and that the signaling through these pathways is intact. TLR2 blockade also inhibited growth of human xenografted tumors in immunodeficient mice. In summary, our data show that TLR2 is a functional receptor expressed in human HNSCC that plays a direct pro-tumorigenic role, and that it can be therapeutically targeted with blocking antibodies to reduce tumor growth.

## INTRODUCTION

Head and neck squamous cell carcinoma (HNSCC) is the most common malignancy of the upper aerodigestive tract and affects 50, 000 Americans each year [1]. It poses a major health problem with a 5-year survival rate of merely 50% in tobacco-related cases [2]. The treatment for HNSCC is based on surgery, radio- and chemo- therapy, but is fraught with frequent treatment failures and complications. While smoking [3], consumption of alcohol [4, 5] and exposure to HPV [6–8] are well-described etiologic factors of head and neck carcinoma, little is known about the molecular mechanisms by which dysplastic lesions progress to carcinoma. Inflammation is gaining recognition as an important driver of tumorigenesis [9, 10], and infection-driven inflammation has been proposed to be involved in 15–20% of human tumors [11, 12]. This is particularly relevant in the case of oral head and neck carcinomas where poor oral hygiene has been linked to increased risk of cancer in several studies [13–15]. Dysplastic mucosal lesions are often super-colonized with microbes, and this colonization has been independently associated with disease progression [15, 16].

The host recognizes invading pathogens via myriad pattern recognition receptors that bind to evolutionarily conserved pathogen-associated molecular pattern motifs (PAMPs) [17]. The Toll-like receptor (TLR) family is a well-described family of membrane-associated proteins that detect and alert the host about incoming pathogens [18–21]. PAMPS such as bacterial wall components, flagella, LPS, ds-RNA, and DNA, bind to TLRs and activate downstream signaling via adaptor molecules MyD88/TRIF with resultant activation of NF-κB and MAPK pathways [22, 23]. TLRs, which are commonly known to be expressed on immune cells [24], orchestrate host responses by leading to maturation of antigen presenting cells, upregulation of co-stimulatory signals, and production of various cytokines and chemokines [25]. It is well understood that TLR stimulation can play an adjuvant role on the immune system and induce anti-tumor responses [26–28]. More recently, however, several TLRs were found to be expressed on a variety of epithelial tissues and epithelial derived tumors [25], and several studies have supported the notion that TLRs may also play a direct pro-tumorigenic role [29–34].

One of the TLR family members, TLR2, is activated by ligands derived from the wall components of gram-positive bacteria and yeast. In oral SCC, which is often colonized with these microbes, TLR2 expression has been described [35, 36]. In particular, several reports have also described an association between oral colonization with yeast and oral epithelial dysplasia [16, 37, 38]. The function of TLR2 in the progression of this malignancy, however, has not been well characterized. Recently, we demonstrated a role for the TLR2/MyD88 pathway in breast and colon epithelial stem/progenitor cell populations [39]. Here, we report that TLR2 is expressed in both HNSCC cell lines and primary patient-derived xenografts and using a yeast-derived agonist, zymosan, we demonstrate that this receptor is functional. Importantly, we show that activation of the receptor has tumor growth-promoting effects, and conversely, antibody targeting of the receptor profoundly inhibits growth.

## RESULTS

### TLR2 is expressed on HNSCC cells and promotes tumor growth

**Figure 1:**
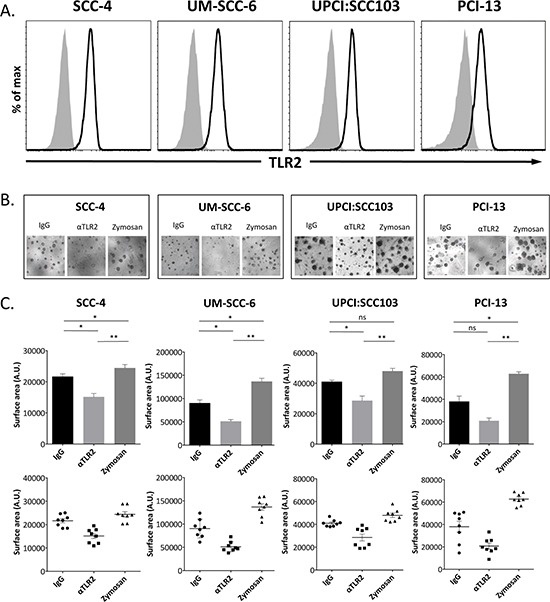
TLR2 is expressed on HNSCC cell lines and promotes growth of organoids in a 3D model of *in vitro* tumor growth **A.** Representative flow cytometry histograms of TLR2 expression (black line) on HNSCC cell lines and isotype-matched control staining (gray shaded histogram). **B.** HNSCC cell lines were grown in organoid cultures in eight replicate wells, and treated with 5 μg/ml mIgG1 antibody, 5 μg/ml α-TLR2 antibody, or 10 μg/ml zymosan. Images shown are representative of cultures at day 14. **C.** Graphs show the mean ± s.e.m. of the total surface area (arbitrary units) of the organoids formed, indicative of cell proliferation, as measured with ImageJ software. Statistical analysis was performed using the Kruskal-Wallis one-way-ANOVA test with Dunn's post-hoc test for multiple comparisons (*n* = 8, *p < 0.05, **p < 0.005, ns = non-significant).

We assessed the expression of TLR2 on established HNSCC cell lines (SCC4, UM-SCC-6, UPCI:SCC103 and PCI-13) by flow cytometry (Fig. 1A), and observed that all four cell lines uniformly expressed the receptor. To determine if TLR2 has a functional role in the growth of HNSCC, we first assessed whether a monoclonal antibody (α-TLR2 mAb, clone T2.5) known to block TLR2 signaling [40], would have any effect on these cell lines in a 3D *in vitro* organoid model of tumor growth. Addition of the α-TLR2 mAb to the organoid cultures resulted in significant reduction of organoid sizes in all cell lines compared to the isotype control (Fig. 1B and 1C), indicating that constitutive activation of the receptor promotes tumor growth. Furthermore, experimental activation of the receptor with a well-characterized yeast-derived ligand of TLR2, zymosan, resulted in a significant increase in the size of the organoids (Fig. 1B and 1C), again indicating that the receptor is functional and has a growth-promoting effect on these cells. Of note, the absolute number of organoids was not consistently affected by the addition of zymosan (Supplemental Fig. 1A), but the individual and aggregate size of the organoids was significantly increased, suggesting that activation of TLR2 may have a profound effect *in vivo*.

**Figure 2:**
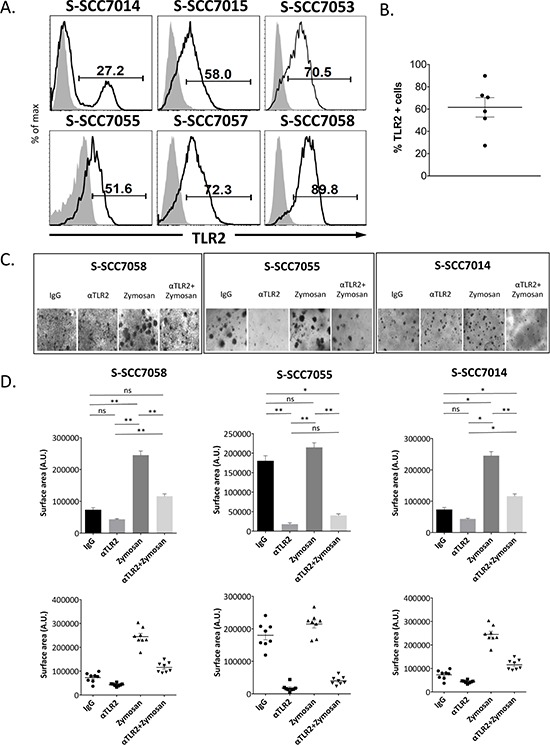
Human primary HNSCC tumors express TLR2, and stimulation of TLR2 with zymosan enhances growth of organoids *in vitro* **A.** Cells from primary human tumors or patient-derived xenografts were isolated and assessed for expression of TLR2 by flow cytometry. **B.** Graph showing % TLR2 positive cells and mean in the primary human tumors. **C.** Primary tumor cells were grown in organoid cultures in eight replicate wells, and treated with 5 μg/ml mIgG1 antibody, 5 μg/ml α-TLR2 antibody, 10 μg/ml zymosan, or both 5 μg/ml α-TLR2 antibody and 10 μg/ml zymosan. Images shown are representative of cultures at day 14. **D.** Graphs show the mean ± s.e.m. of the total surface area (arbitrary units) of the organoids formed, as measured with ImageJ software. Statistical analysis was performed using the Kruskal-Wallis one-way-ANOVA test with Dunn's post-hoc test for multiple comparisons (*n* = 8, *p < 0.05, **p < 0.005, ns = non-significant).

Given these observations with the HNSCC cell lines, we next assessed the expression of TLR2 in patient-derived xenografts (PDX), established from oral squamous cell carcinoma specimens obtained from patients undergoing surgical resection of their tumors. Again, we observed that all of the specimens had a significant proportion of tumor cells with high expression of TLR2, as assessed by flow cytometry (mean 61.8%, s.d. 21.2) (Fig. 2A and 2B). Dissociated cells from three of these PDX tumors reliably formed organoids in 3D cultures by day 14 (Fig. 2C). As observed with the cell lines, exposure of the primary tumor cells to α-TLR2 mAb inhibited organoid growth (Fig. 2C and 2D), indicating that constitutive activation of the receptor was promoting growth in this context. Activation of TLR2 by zymosan resulted in a robust increase in the size of the organoids (Fig. 2C and 2D). Again, the absolute number of organoids was not consistently affected by the addition of zymosan, (Supplemental Fig. 1B), similar to what was observed with the cell lines. Further, the increased growth associated with the addition of zymosan was significantly abrogated by the preincubation and co-culture of the cells with an α-TLR2 mAb, indicating that the effects observed with zymosan were specific to its actions on TLR2. Thus, targeting of TLR2 by the α-TLR2 mAb was able to inhibit both the constitutive and inducible growth-promoting effects of TLR2 in these PDX cells.

### TLR2 signaling through the NF-κB and MAPK pathways is intact in HNSCC and can be blocked by anti-TLR2 antibody

**Figure 3:**
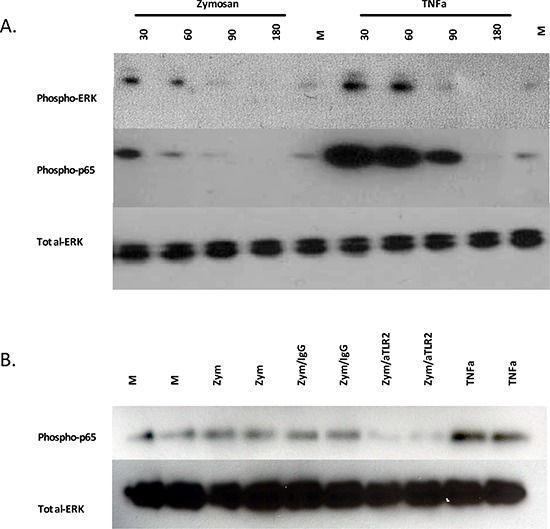
Zymosan activates MAPK/ERK and NF-κB signaling pathways in HNSCC, which can be blocked by anti-TLR2 antibody **A.** UM-SCC-6 cell line was treated with 10 μg/ml zymosan or 10 ng/ml recombinant TNFα for the indicated times and analyzed for expression of phospho-ERK and phospho-NF-κB p65 by Western blots. Total ERK was used as the loading control. **B.** UPCI:SCC103 cells were treated with media alone, 10 μg/ml zymosan, 5 μg/ml mIgG1 antibody and 10 μg/ml zymosan, or 5 μg/ml α-TLR2 antibody and 10 μg/ml zymosan. Cells were collected after 60 min and analyzed for expression of phospho-NF-κB p65 by Western blots.

Because we observed significant inhibition of zymosan-induced tumor growth with an α-TLR2 mAb, we assessed if zymosan could induce the activation of signaling pathways downstream of TLR2. Although activation of NF-κB and MAPK pathways by TLR2 is well described in immune cells, little is known about TLR2 signaling in HNSCC. To investigate this, HNSCC cells were incubated in low-serum conditions with zymosan, and phosphorylation of the p65 subunit of NF-κB and ERK was assessed by Western immunoblot over a time course. Exposure of the cells to zymosan resulted in increased phosphorylation of p65 and ERK at 30 minutes, with levels returning to baseline by 90 minutes after stimulation. Interestingly, the addition of zymosan was able to increase phosphorylation of p65 and ERK above the constitutive baseline (Fig. 3A), consistent with the effects of zymosan on tumor growth in the organoid assays (Fig. 1C and 2D). Notably, incubation of the cells with α-TLR2 mAb was able to inhibit the phosphorylation of p65 (Fig. 3B), confirming that zymosan is acting through TLR2. Together, these data indicate that TLR2 signaling is intact in HNSCC cells and that activation of TLR2 results in the enhanced activation of the NF-κB and MAPK pathways, both of which have previously been shown to be associated with a survival and growth advantage in this malignancy [41, 42].

### Anti-TLR2 antibody inhibits growth of HNSCC tumors *in vivo*

**Figure 4:**
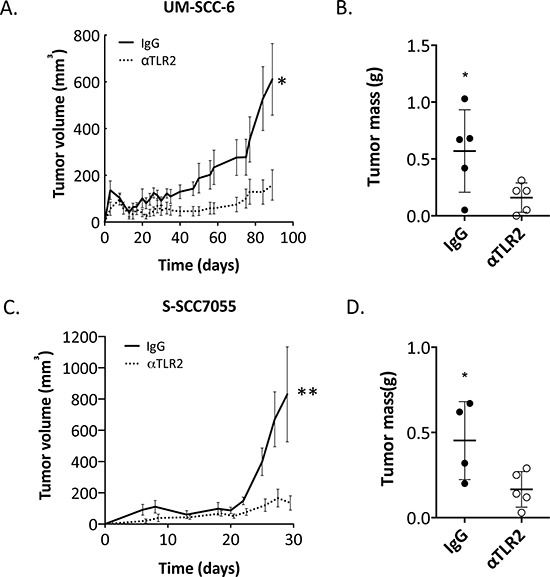
TLR2 blockade inhibits growth of HNSCC tumors *in vivo* **A.** UM-SCC-6 cells were pretreated with 5 μg/ml mIgG1 or 5 μg/ml α-TLR2 antibodies and then treated with 10 μg/ml zymosan for 30 min. Cells were injected subcutaneously into *Rag2^−/−^Il2rg^−/−^* mice and monitored for tumor growth. Graph shows the mean ± s.e.m. of the tumor volumes measured over the course of the experiment, *n* = 5 in each group (*p < 0.05, two-way ANOVA). **B.** Graph shows the mean ± s.e.m. of the tumor wet weights upon termination of the experiment (*p < 0.05, Student's *t*-test). **C.** S-SCC7055 cells were pretreated with 5 μg/ml mIgG1 or 5 μg/ml α-TLR2 antibodies and then treated with 10 μg/ml zymosan for 30 min, as before, and implanted into mice. Graph shows the mean ± s.e.m. of the tumor volumes measured over the course of the experiment, *n* = 4–5 in each group (**p < 0.005, two-way ANOVA). **D.** Graph shows the mean ± s.e.m. of the tumor wet weights upon termination of the experiment (*p < 0.05, Student's *t*-test).

The profound inhibitory effects of α-TLR2 mAb on organoid growth *in vitro* and on TLR2 activation of the NF-κB and MAPK pathways suggested that this might be a viable therapeutic strategy and provided rationale for targeting of TLR2 *in vivo*. To investigate this, UM-SCC-6 cells were pre-treated with either α-TLR2 mAb or isotype control IgG, then subsequently treated with zymosan and implanted into the subcutaneous compartment on the flanks of immunodeficient *Rag2^−/−^Il2rg^−/−^* mice. We observed a substantial reduction in tumor volume and mass in the cohort of mice receiving the α-TLR2 mAb – treated tumor cells (*p* < 0.05, Fig. 4A and 4B). A reduction in tumor volume and mass was also observed in α-TLR2 mAb – treated tumor cells even in the absence of zymosan (Supplemental Fig. 2), indicating the presence of a growth-promoting effect from constitutive TLR2 activation, similar to our *in vitro* studies (Fig. 1C and 2D); however, the antibody-induced reduction in tumor size was not statistically significant. Nevertheless, these data indicated that targeting TLR2 on HNSCC cells inhibits tumor formation *in vivo*.

In a manner similar to the cell lines, dissociated PDX tumor cells were also treated either with α-TLR-2 mAb or isotype control IgG and then subsequently treated with zymosan and implanted into immunodeficient *Rag2^−/−^Il2rg^−/−^* mice. Again, we observed a significant reduction in tumor volume and mass (*p* = 0.04, and 0.04 respectively, Fig. 4C and 4D). Importantly, the single treatment with the α-TLR2 mAb had a durable effect on the growth of these tumors. Thus, targeting TLR2 in HNSCC appears to be an effective strategy to inhibit tumor growth *in vivo*.

## DISCUSSION

In this study, we report that there is significant expression of TLR2 on HNSCC cells and that this receptor is functional with intact signaling through the NF-κB and MAPK pathways. Importantly, the activation of TLR2 confers a growth-promoting effect on these cells, and blockade of the receptor using a monoclonal antibody has a profound inhibitory effect on tumor growth in both *in vitro* organoid and *in vivo* xenograft models.

Our data indicate that there is a constitutive basal and inducible activation of TLR2 in HNSCC. This is based on our findings that treatment with α-TLR2 mAb alone led to a reduction in organoid growth and the observation that there was constitutive phosphorylation of p65 and ERK in these cells. These findings are in line with the known constitutive activation of the NF-κB and MAPK pathways in HNSCC. The activation of these pathways has been associated with the expression of pro-inflammatory and pro-angiogenic factors that can enhance tumor growth [41, 42]. We hypothesize that the activation of these pathways through TLR2 would likely be more pronounced in the context of oral head and neck pre-malignant and malignant lesions that are chronically exposed to large amounts of bacterial and fungal elements.

The role of TLR activation in cancer has been unclear. Expression of the TLR adaptor protein MyD88 has been associated with a more aggressive hepatocellular carcinoma (HCC) phenotype in humans [43, 44], and activating mutations have been found in diffuse large B-cell lymphoma [45]. Moreover we recently showed a cell-intrinsic role for the TLR2/MyD88 pathway in breast and colon epithelial stem/progenitor cell populations, and similar to what we describe here in HNSCC, the inhibition of the TLR2 signaling pathway in breast cancer resulted significant growth inhibition [39]. While there is data that TLR2 has a pro-tumorigenic role in a model of HCC, there is also data pointing to an increased aggressive tumor behavior in HCC in TLR2 deficient mice [46, 47]. In colon carcinoma, activation of TLR9 has been shown to favor survival and chemotherapy resistance of tumor cells [48, 49]. Some of the conflicting results may lie in the fact that the TLRs can have both a role in the immune system and directly on the tumor cells. In the case of oral HNSCC, TLR4 stimulation with LPS has been shown to confer resistance to tumor lysis by natural killer cells and promote survival [49]. These data are supported by studies in non-small cell lung cancer showing that TLR4 stimulation enhances tumor survival *in vitro* and *in vivo* in murine models [50]. In contrast, there are several studies that have implicated an anti-tumorigenic role for TLR signaling. TLR4 single nucleotide polymorphisms with reduced response to LPS have been shown to be associated with a worse prognosis in HNSCC [46]. TLR3 has been shown to inhibit cell growth and directly induce apoptosis *in vitro* in oral head and neck cell lines [46]. Adjuvant effects of TLR stimulation on the immune system are well described and several TLR ligands are currently in use or under investigation as adjunctive anti-tumor therapeutics [20, 51]. Thus, in the planning of therapeutic strategies for targeting the TLRs, it will be very important to consider the direct effects on both the immune system and the tumor cells themselves. Importantly, a humanized IgG4 monoclonal antagonistic antibody, OPN-305, is already in clinical trials for post-transplant delayed graft function [52]. The antibody was able to produce full TLR2 receptor blockade on monocytes and was well tolerated. While it is true that inhibition of the immune response to cancer is a potential concern, there is significant evidence that inflammatory states can be tumor promoting [10]. There is, thus, a need for further investigation to better delineate TLR2′s roles in HNSCC and to determine if it can be manipulated for chemoprevention strategies of high-risk inflammatory associated premalignant lesions.

In conclusion, TLR2 is highly expressed in HNSCC cell lines and primary patient-derived xenografts, and activation of this receptor confers a growth advantage *in vivo* likely through activation of NF-κB and MAPK pathways. These data are of particular importance in the case of oral HNSCC and precancerous lesions where treatment with α-TLR2 antibodies or reduction of the microbial load may have significant effects on disease progression.

## MATERIALS AND METHODS

### Cell lines

The human HNSCC cell line UM-SCC-6 was obtained from Dr. Thomas Carey at the University of Michigan. The SCC-4 cell line was obtained from ATCC. The UPCI:SCC103 and PCI-13 cell lines were kind gifts from Dr. Suzanne Gollin and Dr. Jennifer Grandis at the University of Pittsburgh. Cells were maintained in DMEM/F-12 medium with Glutamax (Gibco, Invitrogen) containing 10% heat-inactivated FBS (Cellgro), 100 IU/ml penicillin and 100 μg/ml streptomycin (Gibco, Invitrogen). The cells were incubated in humidified air with 5% CO_2_ at 37°C, and sub-cultured twice a week with 0.25% trypsin and 0.02% EDTA.

### Primary human tumors and patient-derived xenografts

Tumor specimens were procured through the Stanford University Tissue Bank, through a protocol approved by the Institutional Review Board. Dissociated tumor cells were implanted into the subcutaneous compartment of *Rag2^−/−^Il2rg^−/−^* immunodeficient mice, in accordance with the Stanford University Administrative Panel on Laboratory Animal Care. Briefly, mice were anesthetized with isoflurane-oxygen, and cells immersed in Matrigel (BD, Pharmingen) were injected subcutaneously into the flanks of mice. Mice were monitored for tumor growth, and xenograft tumors were harvested when they reached a maximum size of 1 cm in diameter.

### Tumor digestion

Tumors were mechanically and enzymatically dissociated and digested in a solution of 300 U/ml collagenase and 100 U/ml hyaluronidase (StemCell Technologies) in culture media; DMEM/F-12 with 5% FBS, 1% penicillin-streptomycin, 1 × penicillin-streptomycin-amphotericin B (MP Biomedicals), and 25 mM HEPES, at 37°C for 3 h. The dissociated cells were spun down and resuspended in Trypsin-EDTA (StemCell Technologies) for 3 min, then further dissociated with pre-warmed 5 mg/ml dispase (StemCell Technologies) and 0.1 mg/ml DNase I (StemCell Technologies) for 1 min. Cells were filtered through a 40 μm cell strainer, washed with culture media and used for assays.

### Flow cytometry analysis

Cells were stained with a panel of antibodies in PBS containing 2% FBS, 1% penicillin-streptomycin, and 1 mM EDTA. Tumor cells from primary tumor samples were identified by gating out leukocytes, endothelial, and stromal cells expressing human lineage markers: CD2 (RPA-2.10), CD3 (UCHT1), CD18 (6.7), CD31 (WM59), CD45 (HI30), and CD64 (10.1). Tumor cells from xenografts passaged in mice were identified by gating out stromal cells expressing mouse lineage markers: H-2K^d^ (SF1–1.1), H-2K^b^ (AF6–88.5) and muCD45 (30–F11). Anti-CD31 was obtained from eBioscience, and all other antibodies were obtained from BD Pharmingen. All lineage antibodies were biotinylated or directly conjugated to PE/Cy5. Labeled cells were then washed and stained with streptavidin-PE/Cy5 (BioLegend). DAPI was obtained from Invitrogen.

After gating out leukocytes, endothelial, stromal, and non-viable cells, the tumor cells were profiled for expression of TLR2. Anti-TLR2 (T2.5) was obtained from eBioscience. Cells were analyzed on a BD LSRFortessa. Events collected were analyzed using FlowJo Version 9.6.4 software (Tree Star).

### Reagents and blocking antibodies

For the TLR2 blocking experiments, tumor cells were treated with 5 μg/ml α-TLR2 (T2.5, BioLegend, eBioscience), or 5 μg/ml mIgG1 isotype control (MOPC-21, BioLegend) antibodies. For the organoid assays and *in vivo* tumor assays, the TLR2 ligand zymosan was obtained from Novus Biologicals (NBP2–26233). For the western blots, zymosan was obtained from Santa Cruz (sc-258367). Zymosan was added to assays at a concentration of 10 μg/ml.

### Organoid assay

Organoids were grown using a protocol adapted from Sato et al. [53] Briefly, Wnt3A-cells (irradiated L1 feeder cells) were plated in non-tissue culture 96-well plates in DMEM/F-12 media with 10% FBS and 1% penicillin-streptomycin. Cells were allowed to attach for 2 h before 50 μl of growth factor reduced Matrigel (BD Biosciences) was added on top. Thereafter, 5000 cells resuspended in 100 μl of culture media were added on top of the Matrigel and feeder cells in each well.

For the cell lines, the culture media used was DMEM/F-12 medium with 10% FBS and 1% penicillin-streptomycin. For primary human tumors and patient-derived xenografts, Clevers media was used, which comprised DMEM/F-12 medium, 10 mM HEPES, 1 × Glutamax, 10% FBS, 1 × N-2 supplement (Gibco), 1 × B-27 supplement (Gibco), 500 ng/ml human R-Spondin 3 (R&D Systems), 10 ng/ml human EGF (PeproTech), and 100 ng/ml human Noggin (PeproTech).

Organoids were grown in humidified tissue culture incubators at 37°C in 5% CO_2_ and 20% O_2_ and monitored daily under a microscope. Media supplemented with 5 μg/ml α-TLR2 antibody, 5 μg/ml mIgG1 antibody, or 10 μg/ml zymosan was changed twice a week.

### Measurements of organoid formation

Organoids were cultured in eight replicate wells for each treatment condition, and one image from each well was photographed on day 14 of culture. The images were analyzed by ImageJ software (NIH) to determine the total area and number of organoids formed.

### SDS electrophoresis and western blotting

Cells were plated onto 12- or 24-well plates, and treated with 5 μg/ml of antibodies for 1 h. After 1 h, 10 μg/ml of zymosan was added. Cells were lysed by adding cold 1 × SDS loading buffer into the wells. Cell lysates were collected, boiled for 5 minutes, and loaded in precast 4–12% gradient gels (Life Technologies). After separation of proteins via electrophoresis, proteins were transferred to PVDF membranes. Blots were blocked with 5% milk in TBST (Cell Signaling) for 1 h. Primary detection antibody was diluted in 5% BSA and TBST for phospho blots and 5% milk and TBST for non-phospho blots. Secondary goat-anti-mouse or anti-rabbit antibodies conjugated to HRP were used for chemiluminescent detection with WesternBright ECL HRP substrate (Advansta). Phospho-ERK antibody (E-4, sc-7383), and ERK 2 antibody (K-23, sc-153) were obtained from Santa Cruz Biotechnology. Phospho-NF-κB p65 (Ser536) (93H1, 3033S) was obtained from Cell Signaling Technology.

### *In vivo* xenograft model

Cells were pretreated with 5 μg/ml of α-TLR2 or mIgG1 isotype control antibodies for 30 min. Where stated, cells were then treated with 10 μg/ml zymosan for an additional 30 min before implantation. Viable cells were counted and (5 × 10^6^ for UM-SCC-6, 10 × 10^6^ for UPCI:SCC-103 and 2 × 10^6^ for S-SCC7055) injected subcutaneously into each animal. Groups of 5 mice were used for all experiments except for S-SCC7055, where 4 mice were used in the control group. Tumor growth was assessed using calipers three times per week. Mice injected with UPCI:SCC103 and S-SCC7055 cells were euthanized before the 4^th^ week due to tumor size. The length and width of the tumors were measured, and tumor volume was calculated according to the following formula: Tumor Volume = Length × (Width)^2^.

### Mice

B10;B6–*Rag2^−/−^II2rg^−/−^* mice (Taconic) and BALB/c-*Rag2^−/−^II2rg^−/−^* mice (The Jackson Laboratory) were bred and maintained under specific pathogen-free conditions. Mice at 6–12 weeks were used for experiments. All procedures were performed in accordance with protocols approved by the Administrative Panel on Laboratory Animal Care at Stanford University.

### Statistical analyses

Statistical analysis comparing the total number of organoids between different conditions was performed using the Student's *t*-test. The Kruskal-Wallis one-way-ANOVA test with Dunn's post-hoc test for multiple comparisons was used for comparing the total area measurements in the organoid assays.

## SUPPLEMENTAL FIGURES


