# Supplementary material for: Targeting Toll-like receptor 2 inhibits growth of head and neck squamous cell carcinoma
Source: Oncotarget. 2015 Apr 2;6(12):9897–907. doi: 10.18632/oncotarget.3393 (PMC4496405; doi:10.18632/oncotarget.3393)
Supplement: Supplementary file 1 [file oncotarget-06-9897-s001.pdf]

SUPPLEMENTAL FIGURES

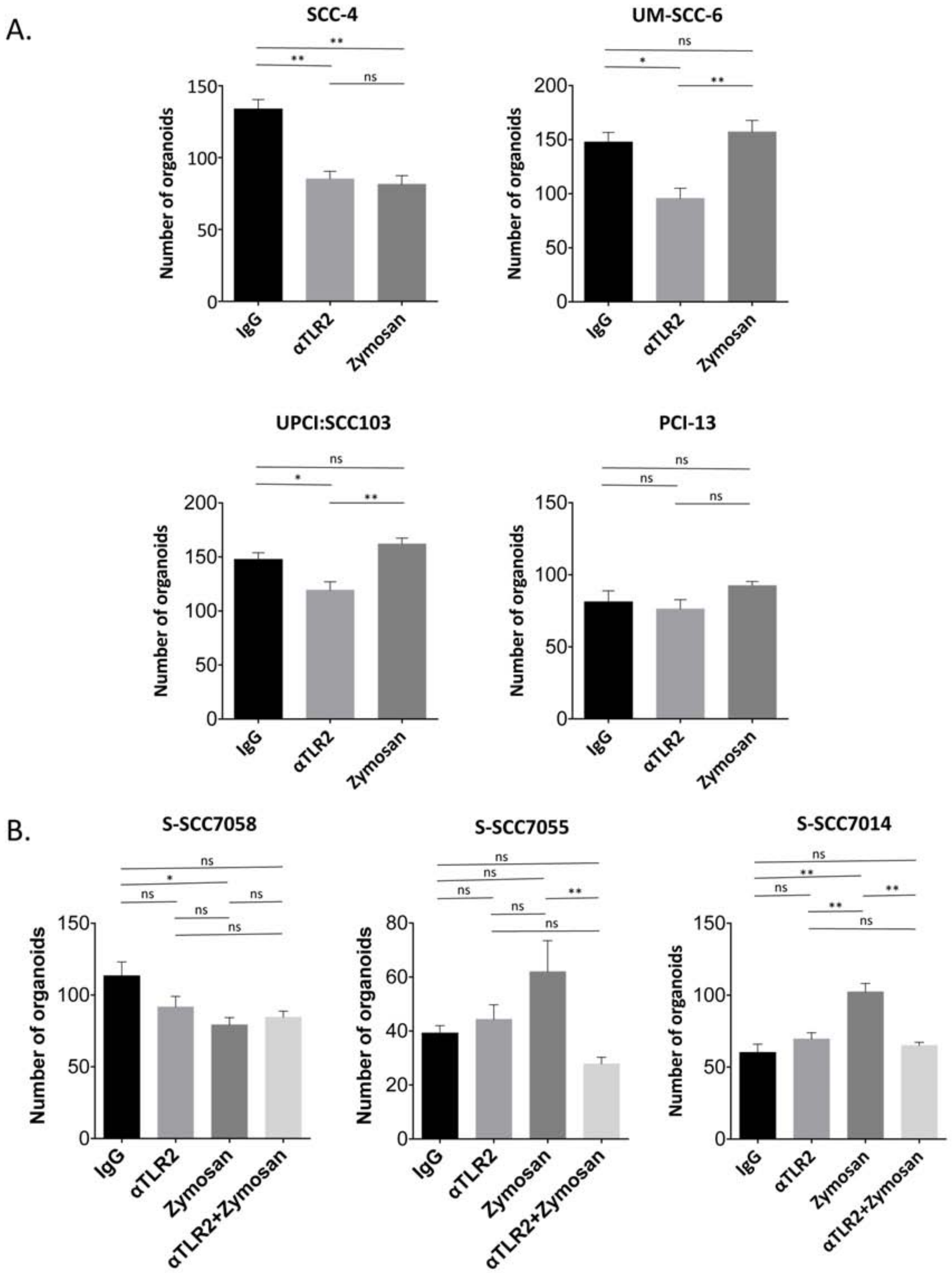

Supplementary Figure S1: Effect of TLR2 on organoid formation. Graphs show the mean ± s.e.m. of the total number of organoids formed, as measured with ImageJ software for **A.** cell lines and **B.** patient-derived xenografts. Statistical analysis was performed using the Kruskal-Wallis one-way-ANOVA test with Dunn's post-hoc test for multiple comparisons ($n = 8$, *$p < 0.05$, **$p < 0.005$, ns = non-significant).

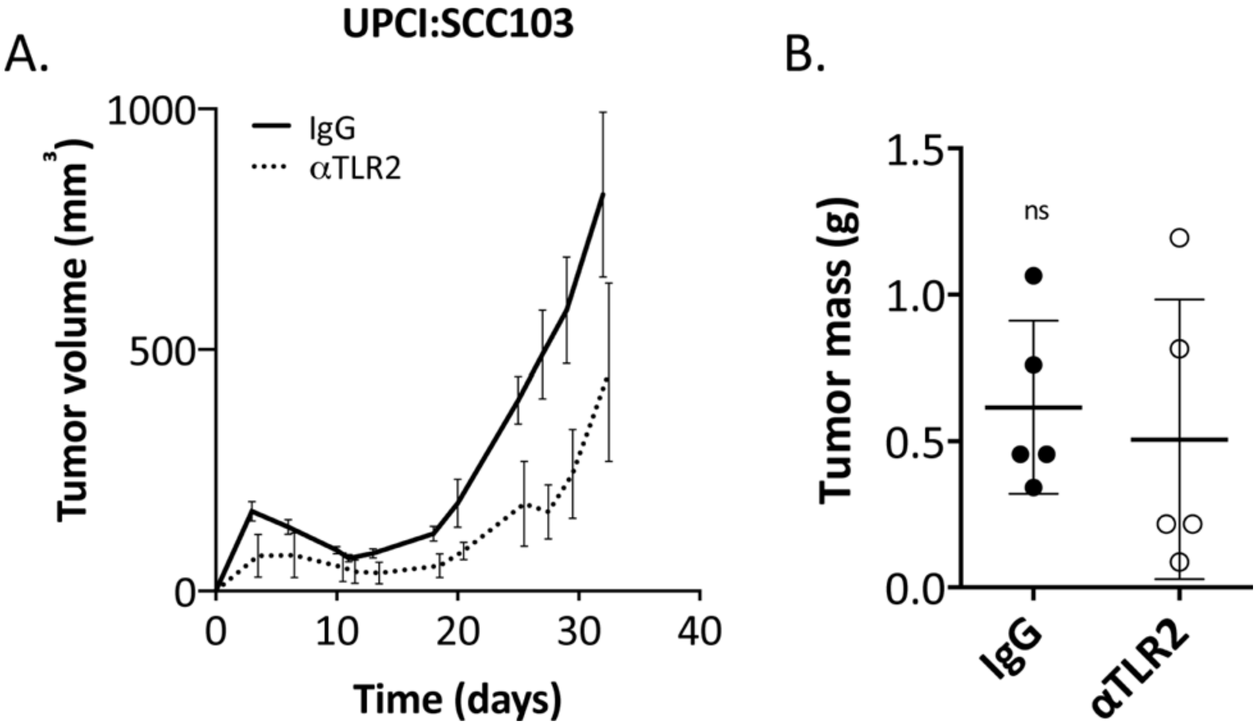

Supplementary Figure S2: TLR2 blockade inhibits growth of HNSCC tumors *in vivo*. A. UPCI:SCC103 cells were pretreated with 5 µg/ml mIgG1 or 5 µg/ml α-TLR2 antibodies and then injected subcutaneously into *Rag2*$^{-/-}$*Il2rg*$^{-/-}$ mice. Graph shows the mean ± s.e.m. of the tumor volume measured over the course of the experiment, *n* = 5 in each group (ns = non-significant, two-way ANOVA). **B.** Graph shows the mean ± s.e.m. of the tumor wet weight upon termination of the experiment (ns = non-significant, Student's *t*-test).
